# Interpreting Pregnancy Risk After GLP‐1 Receptor Agonists Discontinuation

**DOI:** 10.1111/dom.70615

**Published:** 2026-02-26

**Authors:** Roberta Scairati, Christine Newman, Fidelma P. Dunne, Annamaria Colao

**Affiliations:** ^1^ Dipartimento di Medicina Clinica e Chirurgia, Sezione di Endocrinologia, Diabetologia, Andrologia e Nutrizione Università Federico II di Napoli Naples Italy; ^2^ HRB Clinical Research Facility Galway, University of Galway Co Galway Ireland; ^3^ Institute for Clinical Trial, Clinical Science Institute, University of Galway Galway Ireland; ^4^ UNESCO Chair for Health Education and Sustainable Development, Federico II University Naples Italy

**Keywords:** gestational diabetes, GLP‐1 analogue, obesity care, pregnancy, weight management

Glucagon‐like peptide‐1 receptor agonists (GLP‐1RAs) have rapidly revolutionised the management of obesity and type 2 diabetes, including among women of reproductive age. Although these drugs are contraindicated in pregnancy, clinicians are navigating a growing number of women who conceive shortly after treatment discontinuation, in the virtual absence of specific randomised evidence to guide counselling and clinical management. This emerging scenario challenges traditional approaches to metabolic risk assessment in pregnancy and calls for a more dynamic framework.

Recent large real‐world analyses have suggested that pregnancies preceded by GLP‐1RAs exposure are associated with greater gestational weight gain and higher risks of gestational diabetes, hypertensive disorders of pregnancy and preterm birth compared with unexposed pregnancies with similar pre‐pregnancy body mass index (BMI), while rates of caesarean delivery and extremes of birth weight do not differ appreciably [1]. These findings do not establish causality, but they sharpen risk stratification and justify closer attention to gestational weight gain and cardiometabolic complications in this increasingly common scenario. At the same time, a different and equally pressing question for preconception care emerges: for women with severe obesity and high baseline metabolic risk, does treatment with a GLP‐1RA before pregnancy ultimately improve or worsen perinatal outcomes compared with never treating? Addressing this requires moving beyond static measures of BMI towards the concept of *weight trajectories* across the periconception period.

GLP‐1RAs are typically prescribed to individuals with long‐standing, often severe obesity and metabolic dysfunction. In practice, their use is increasingly extending to women with overweight (BMI 25–30 kg/m^2^), including prescriptions within fertility services, groups that are largely invisible in available datasets but frequently exposed to subsequent pregnancies [2, 3]. On treatment, many experience substantial weight loss with improvements in glycemic and blood pressure control, while after discontinuation, weight regain is common, a pattern of weight cycling documented in randomised trials and real‐world follow‐up [4, 5, 6]. When this sequence of weight change overlaps with the periconception or early pregnancy window, discontinuation of GLP‐1RAs may trigger a metabolic whiplash, a rapid shift from pharmacologically assisted weight loss back towards the underlying obesity and insulin‐resistant phenotype precisely when gestational weight trajectories are being set. Matching on BMI near conception improves comparability and makes the clinical message easy to read, but inevitably strips away key elements of the trajectory. It obscures starting weight before GLP‐1RAs use, the magnitude and rate of on‐treatment weight loss, the speed of weight regain after discontinuation, and the alignment of these dynamics with conception. In this context, BMI at conception represents only a single frame in a much longer film of weight gain and loss. Reliance on a single periconception BMI measure can effectively erase much of the pre‐treatment risk profile and weaken inference about net benefit. Optimising metabolic health before pregnancy is therefore a central goal of preconception care, but its evaluation requires attention to how weight and metabolic risk evolve over time, not solely where they intersect with conception [7, 8]. Beyond weight dynamics, GLP‐1RAs lower postprandial glycemia in part by delaying gastric emptying, thereby slowing intestinal glucose delivery and blunting early glucose excursions [9, 10]. Treatment discontinuation removes this constraint, accelerating gastric emptying and amplifying early postprandial glucose peaks. In the periconception window, such rebound may exaggerate glycemic responses, potentially contributing to gestational diabetes diagnoses independently of major changes in adiposity. This shift in glucose kinetics may represent an overlooked component of post‐discontinuation metabolic instability.

An additional, and often under‐appreciated, dimension of this trajectory is diet. Habitual dietary patterns before GLP‐1RAs initiation, changes during pharmacologically assisted weight loss, and diet quality in early pregnancy are not reported, and they are rarely retrievable from electronic health records. This is a critical blind spot. Across study designs, evidence indicates that dietary patterns characterised by higher intakes of minimally processed, plant‐forward foods, including Mediterranean‐style and low‐glycemic index approaches, are associated with modest reductions in gestational weight gain and improved glycemic control in women at high metabolic risk, with no clear adverse effects on neonatal outcomes [11, 12, 13, 14, 15, 16]. Diet is therefore not a marginal covariate but a core driver of any weight‐trajectory phenotype. Among women who discontinue GLP‐1RAs around conception, trajectories are likely to diverge. Some may maintain a high‐quality, structured diet and limit weight regain, whereas others may revert to energy‐dense, nutrient‐poor patterns that facilitate rapid regain and destabilise glycemia. Although dietary quality is modifiable, its reliable assessment and sustained support across preconception and pregnancy are challenging in routine care, often requiring specialised expertise and repeated follow‐up. As a result, these dimensions remain largely unmeasured and cannot be adjusted for in the propensity score, leaving room for residual and potentially substantial confounding. More importantly, from a clinical perspective, diet is precisely where the potential to counterbalance metabolic whiplash is greatest.

Extending the distinction proposed between pre‐treatment and periconception BMI, we propose a multidimensional weight‐trajectory phenotype that integrates four axes: baseline obesity severity and metabolic risk before treatment, the magnitude and rate of GLP‐1RAs‐induced weight loss, diet and lifestyle quality before and during pregnancy, and the speed and extent of weight and glycemic rebound after treatment discontinuation. Linked data across obesity and diabetes management, primary care and obstetrics, paired with explicitly designed target‐trial emulations, could then compare women with similar starting profiles who follow different trajectories through the periconception period. Such an approach would allow investigators to quantify how distinct patterns of weight change relate to gestational weight gain, gestational diabetes, hypertensive disorders, preterm birth and offspring outcomes, and could prevent underestimation of potential benefits when GLP‐1RAs are used strategically before pregnancy. While awaiting such data, the implications for clinical practice can already be refined. A recent history of GLP‐1RAs use should prompt a structured review of pre‐treatment weight, weight change on and off therapy, and dietary counselling, alongside early attention to glycemic control and blood pressure. This should not be interpreted as evidence that GLP‐1RAs exposure is intrinsically harmful. Rather, preconception prescribing should be paired with pregnancy planning, reliable contraception and a proactive transition strategy.

Beyond intensified dietary and behavioural support, one pragmatic option is the use of metformin in the preconception period and early pregnancy. Randomised trials and meta‐analyses in obese and high‐risk women suggest that metformin can limit gestational weight gain and improve glycemic stability without clear adverse neonatal effects, even in the absence of overt diabetes [17, 18, 19]. In addition to these established effects, metformin delays gastric emptying and attenuates postprandial glycemic excursions through mechanisms that partly overlap with those of GLP‐1RAs [20, 21]. Following GLP‐1RA discontinuation, when gastric emptying may accelerate and destabilise glucose dynamics, this effect may help moderate postprandial excursions and smooth the return to underlying insulin‐resistant physiology. Taken together, these properties provide a clear rationale for metformin as a pharmacologic bridge to attenuate post‐discontinuation weight regain and metabolic rebound while remaining compatible with pregnancy. As an inexpensive and globally available therapy, metformin represents a feasible strategy that warrants further evaluation in this context, particularly given the rapid expansion of GLP‐1RAs exposure before pregnancy beyond traditional indications.

Collectively, once pregnancy occurs after recent GLP‐1RAs discontinuation, it appears prudent to obtain a structured history of pre‐treatment weight, subsequent weight changes and diet quality, and to adopt a low threshold for early and repeated assessment of glycemia and blood pressure, in line with current standards of care [22].

An essential discussion has emerged on how to counsel and care for women who use GLP‐1RAs and later become pregnant. The next step is to define which weight‐trajectories are protective and which carry a rebound penalty around conception, aligning monitoring and support accordingly. Moving beyond BMI snapshots towards weight‐trajectories phenotypes will be key to judging, with appropriate nuance, when preconception GLP‐1RAs therapy represents an opportunity to improve reproductive outcomes and when careful transition planning is required.

## Author Contributions

R.S. researched data, contributed to discussion and wrote the first draft of the manuscript. R.S., C.N., F.P.D. and A.C. reviewed and edited the manuscript, providing critical intellectual insights. All the authors approved the final version of the study.

## Funding

The authors have nothing to report.

## Conflicts of Interest

The authors declare no conflicts of interest.

## Data Availability

Data sharing not applicable to this article as no datasets were generated or analysed during the current study.
